# Comparison of cytokine/chemokine levels in aqueous humor of primary open-angle glaucoma patients with positive or negative outcome following trabeculectomy

**DOI:** 10.1042/BSR20181894

**Published:** 2019-05-02

**Authors:** Beata Gajda-Deryło, Thomas Stahnke, Stephan Struckmann, Gregor Warsow, Kerstin Birke, Marco T. Birke, Bettina Hohberger, Robert Rejdak, Georg Fuellen, Anselm G. Jünemann

**Affiliations:** 1 Department of General Ophthalmology, Medical University in Lublin, Lublin, Poland; 2 Department of Ophthalmology, Rostock University Medical Center, Rostock, Germany; 3 Institute for Biostatistics and Informatics in Medicine and Ageing Research, Rostock University Medical Center, Rostock, Germany; 4 Department of Ophthalmology, Tufts University Boston, MA, U.S.A.; 5 Department of Ophthalmology, Friedrich-Alexander University Erlangen-Nuremberg, Erlangen, Germany

**Keywords:** bioinformatics, ophthalmology, protein expression

## Abstract

We aimed to identify differences in cytokine/chemokine levels in the aqueous humor (AH) of primary open-angle glaucoma (POAG) patients who suffered from scarring, compared with POAG patients with no scarring after trabeculectomy surgery. Identification of differently expressed cytokines and chemokines may help to understand scarring and fibrotic processes following trabeculectomy, and to make predictions for the outcome of fistulating surgery in the future. Furthermore, the identification of cell signaling pathways involved in fibrosis offers the opportunity for a more specific antifibrotic therapy with reduced side effects, and an improvement in long-term surgical outcome.

Eight samples of AH were collected during trabeculectomy surgery and commercially available cytokine/chemokine arrays were used. Specific, differently expressed proteins (cytokines/chemokines) in AH samples from patients with positive and negative surgery outcomes were detected. These proteins were classified based on their known profibrotic, inflammatory, adhesive, and apoptotic properties. Transforming growth factor β (TGF-β) and vascular endothelial growth factor (VEGF) were among the most important profibrotic cytokines that we detected. Differences in the fold change of protein expression were highly significant between patients after successful and failed trabeculectomy surgery, and these were processed and visualized using *ExprEssence* software.

This pilot study revealed differences in concentrations of cytokines/chemokines in AH between the two examined groups of patients. Our findings suggest that a positive outcome from trabeculectomy is strongly related to an inhibition of the fibrosis process.

## Introduction

Glaucoma is the second leading cause of irreversible blindness worldwide [1]. It is a neurodegenerative disease accompanied by the degeneration of retinal ganglion cells (RGCs) and their axons. Patients with glaucoma present with loss of central vision and of the visual field [2]. The most common type of glaucoma is primary open-angle glaucoma (POAG), which can occur with elevated or normal intraocular pressure (IOP). Inequality in production of aqueous humor (AH) and its drainage through the trabecular meshwork leads to increased IOP levels [3,4]. Increased IOP levels are discussed as the main risk factor for glaucoma progression [5].

Treatment of glaucoma patients usually starts with eye drops reducing AH production or increasing AH drainage. Surgical procedures are performed among those patients whose topical treatment is insufficient, who develop allergic reactions, or are not in the condition to applicate eye drops. Trabeculectomy is known from the 1960s and is still one of most preferable glaucoma filtration surgeries [6]. Its purpose is to create an outflow channel for AH that connects the anterior chamber (AC) with the sub-Tenon’s space [6]. After trabeculectomy, wound healing processes often lead to fibrosis and scar formation at the site of the new drainage which disrupts AH outflow [6,7]. The prolonged presence of myofibroblasts producing α-smooth muscle actin (α-SMA) plays a key role in scar formation that leads to unwanted wound closure [8]. This process is stimulated by growth factors and inflammation [9]. Extracellular matrix (ECM) proteins like fibronectin and collagens are also produced by activated myofibroblasts [10].

Matrix metalloproteinases (MMPs) are proteolytic enzymes whose function is to reduce the number of ECM proteins during ECM remodeling. They are dysregulated in many diseases with excessive scar formation. Down-regulation of MMPs leads to disruption between synthesis and degradation of ECM components [7].

Many experiments demonstrated increased levels of different proteins in the AH of patients with POAG [11–13]. The most important growth factors/cytokines among them, which play an indispensable role in fibrotic processes, are transforming growth factor β (TGF-β), vascular endothelial growth factor (VEGF) and tumor necrosis factor-α (TNF-α). Additionally, members of the interleukin family take a central role in fibrosis. Interleukins with levels distinctly elevated in POAG patients are IL-6 and IL-8 [14,15].

*ExprEssence*, which we employ as the main post-processing and visualization tool for our protein expression data, has previously been used on gene expression (transcriptomics) data from kidney [16], pluripotency [17], and breast cancer [18], among others. The software can naturally be applied to protein expression data as well. The characteristic feature of *ExprEssence* is its focus on known protein interactions as a guide to select specifically informative changes of gene or protein expression. A frequently used and freely available source of gene–protein and protein–protein interaction data in the form of a network is the STRING database [19].

The aim of this pilot study was to compare proteins from the AH of positive (no fibrosis) versus negative (fibrosis) early outcomes of POAG patients who were surgically treated by trabeculectomy. A differential analysis of protein expression was performed based on a STRING network using *ExprEssence*, to identify protein interactions and signaling pathways involved in fibrosis. The differences in expression levels, mapped on to a network of protein interactions, specifically for growth factors/cytokines, sheds some light on to fibrotic mechanisms following trabeculectomy, and these may partially explain its failure in some patients but not others.

## Materials and methods

### Ethics statement

The Institutional Review Board of the University of Erlangen-Nürnberg approved this case–control study. The purpose and methods in the present study were explained in detail to patients before participating. Written and informed consent was obtained from the patients. The present study was carried out in accordance with the tenets of the Declaration of Helsinki.

### Patients and design

All participants presented to the Department of Ophthalmology at the University of Erlangen-Nuremberg for trabeculectomy. Criteria for POAG were an open anterior chamber angle, glaucomatous changes of the optic nerve head, and an elevated IOP (>21 mmHg). Indications for trabeculectomy were an IOP under maximal medication exceeding the target pressure or progression of glaucomatous damage. Patients with local side effects of antiglaucomatous medications as indication for trabeculectomy were not included. Only eyes without previous ocular surgery were included. All eyes were phakic. One week before surgery, the local antiglaucomatous medications were taken off and local steroids without preservative were given five times a day. The IOP was controlled by systemic carbonic anhydrase inhibitors in all patients.

Criteria for early failure of trabeculectomy was the need for bleb needle revision two times within the first 3 months after trabeculectomy and an IOP > 21 mmHg without medications, 3 months after trabeculectomy. Criteria for early success of trabeculectomy was IOP < 13 and > 8 mmHg without medications, 3 months after trabeculectomy.

Trabeculectomy was performed by one surgeon (A.G.J.) under topical gel anesthesia. The eye was fixed using corneal traction suture. The following surgical steps were performed: (i) limbal opening of the conjunctiva from 11 to 1 o’clock, (ii) opening and hydrodissection of the tenon, (iii) application of mitomycin C (0.03% for 3 min) using a butterfly-shaped sponge and subsequent wash-out, (iv) creation of a rectangular scleral flap 4 by 4 mm, (v) preplacement of two 10/0 nylon sutures at the posterior edges of the scleral flap, (vi) paracentesis at 2 o’clock position, (vii) rectangular corneoscleral excision 2 by 1 mm including Schlemm’s canal and trabecular meshwork, (viii) basal iridectomy, (ix) closure of the flap sutures after refilling the anterior chamber using balanced salt solution, (ix) readapting the tenon at the limbus using two 10/0 nylon sutures, (xi) readapting the conjunctiva at the limbus using two 10/0 nylon sutures and (xii) removal of the corneal traction suture.

Patients with positive and negative outcomes were matched by age, duration of glaucoma, preoperative IOP, stage of the glaucoma and number of local antiglaucomatous medications. Two patients with positive outcome (nr 2 and 3) and one patient with negative one (nr 6) received local preservative-free steroids preoperatively for 1 week. An *a priori* power analysis based on preliminary data showed that a sample size of 3–5 per group should be sufficient for detecting significant differences in protein levels in the AH.

Thus, out of the patients undergoing trabeculectomy between June 2009 and July 2009 and for whom samples of AH were available, a total of eight POAG patients met the inclusion criteria and could be admitted into the study. Three of them matched the inclusion criteria for early failure by fibrosis; 1 male, 2 female, mean age: 61 ± 11, and 5 of them matched the inclusion criteria for early success with no fibrosis; 4 females, 1 male, mean age: 53.8 ± 8.2. All participants were of Caucasian race, with an average age of 59 (± 9.44–74) years. Patient characteristics are given in Table 1. There was no statistical difference in age, number of preoperative medications, duration of glaucoma, MD and preoperative IOP between the two groups of patients.

**Table 1:** Clinical characteristics of patients

	Age	Gender	Trab outcome	Type of med	Number of med*	Stage of OA	MD	IOP
**1**	62	F	+	timolol, brimonidine	2	2	1.1	14
**2**	46	F	+	timolol	1	4	11.4	15
**3**	58	F	+	timolol	1	3	2	35
**4**	44	F	+	acetazolamide, latanoprost, brinzolamide	3	3	4.7	24
**5**	59	M	+	acetazolamide, brimonidine, timolol, brinzolamide	4	1	3.4	28
**6**	74	F	-		0	3	−0.3	26
**7**	54	F	-	tImolol, acetazolamide, tafluprost,	3	3	−0.4	17
**8**	55	M	-	latanoprost, timolol	2	1/2	1.7	11

Abbreviations: med, antiglaucomatous medication; Trab, trabeculectomy. * = Number of medications before they were taken off 1 week before surgery, stage of OA: glaucomatous optic nerve atrophy according to the stages given by Jonas et al. [20], MD, mean deviation in standard automated perimetry (Octopus 500).

### Laboratory analysis

AH samples were obtained intra-operatively by A.G.J. prior to the opening of the conjunctiva. A total of 100–150 µl of AH was withdrawn through an *ab externo* limbal paracentesis site using a 27-gauge needle on a tuberculin syringe, with special care to avoid blood contamination. The samples were immediately frozen in liquid nitrogen and stored in a deep freezer at −80°C until biochemical analysis. A total of 274 different proteins in total were analyzed in each sample by Cytokine Antibody arrays (RayBio Cytokine Antibody Array C Series 4000; RayBiotech, Inc, Norcross GA 30092, U.S.A.). Antibody arrays were used according to the manufacturer’s protocol. Briefly, after an initial blocking step, 50 μl of each aqueous sample was incubated on each membrane overnight at 4°C. Antibodies provided by the company were used to detect the protein levels. Signals were visualized by exposure to light-sensitive films (Hyperfilm ECL; GE Healthcare, Munich, Germany), which were digitized and densitometrically quantitated with the Multi Gauge V3.1 software (Fujifilm, Düsseldorf, Germany), giving rise to 274 protein expression measurements per sample. Supplementary Data S1 (in Excel format) includes the data of the five Cytokine Antibody arrays covering the 274 proteins (excl. positive, negative and blank controls), with two measurements per patient, that is, ten measurements for the five patients without fibrosis, and six measurements for the three patients with fibrosis.

### Statistical analysis

Statistical differences between AH protein levels in patients with and without fibrosis were assessed using the two-way Anova test and Bonferroni correction for multiple comparisons. Statistical analyses were performed using GraphPad Prism Software (GraphPad, Inc. CA, U.S.A.). The significance level was established at *P*≤0.05. Data are presented as mean ± standard deviation (S.D.).

### ExprEssence analysis

ExprEssence was applied in version 1.2 within Cytoscape 2.6.0 on a human network downloaded from string-db.org in version 9.1. The experimental score threshold was set to 0.8, the other scores (database and textmining) were set to 0.9, yielding a network comprising 27042 interactions (18685 after removal of redundant interactions stemming from different sources) between 6577 proteins in total. The 274 protein expression measurements were then mapped on to the network using the protein names as common identifiers, yielding a network comprising 358 interactions between 199 proteins. The *ExprEssence* LinkScore (see next paragraph) was mapped on to the links (the interactions between the proteins) and is shown by width of the link as well as a red-green color gradient.

Setting the LinkScore-threshold of the quantile-based sliders to 10 and 90%, we condensed the starting network provided by STRING to 70 interactions between 64 proteins. The condensed network was then analyzed manually.

**Figure 1:**
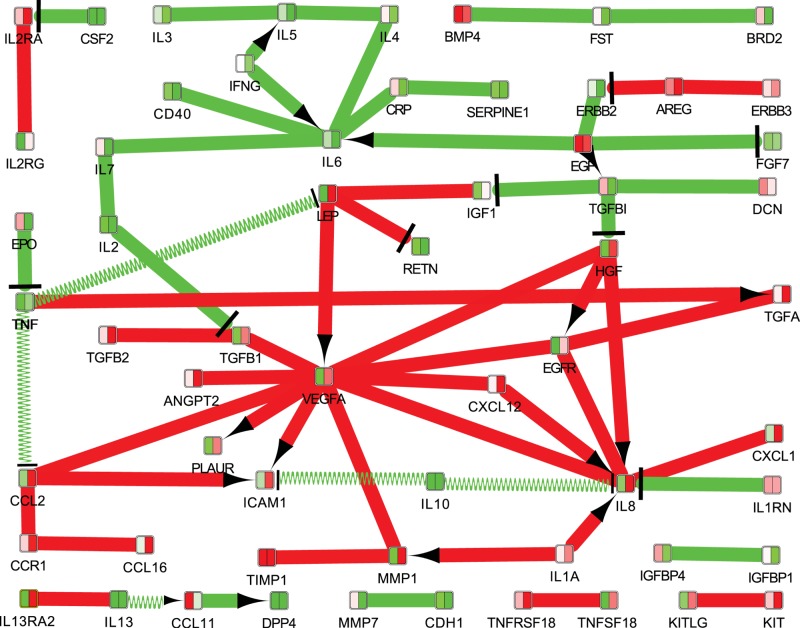
Fibrotic mechanisms following trabeculectomy, visualized based on mapping the protein expression data on to a STRING network Edges with LinkScores close to zero were removed, keeping only 10% of the edges with the largest and 10% of the edges with the smallest LinkScores. The LinkScore is represented by color (green = small, red = large) and by the thickness of the links. Inhibition links are drawn with a T-bar, activation links with an arrow. Interaction links without known direction are shown without T-bar or arrow. The colors in the nodes represent the protein expression measurements for the two conditions (left = healthy, right = fibrotic).

ExprEssence calculates a score for each undirected interaction and for each activating interaction as the sum of the logarithmic fold changes of the protein expression values. For inhibiting interactions, the score is the difference of the logarithmic fold changes. The network is then condensed by removing all interactions that fail to score above or below a given threshold (in a quantile-based fashion), see Figure 1.

Supplementary Data S2 (in Cytoscape format) includes the data of the Cytokine Antibody arrays covering the 274 proteins, mapped on to the String network and condensed by *ExprEssence*, in the view ‘condensed Network 1 (positive − negative)’, which was used to generate the figure.

## Results

**Figure 2:**
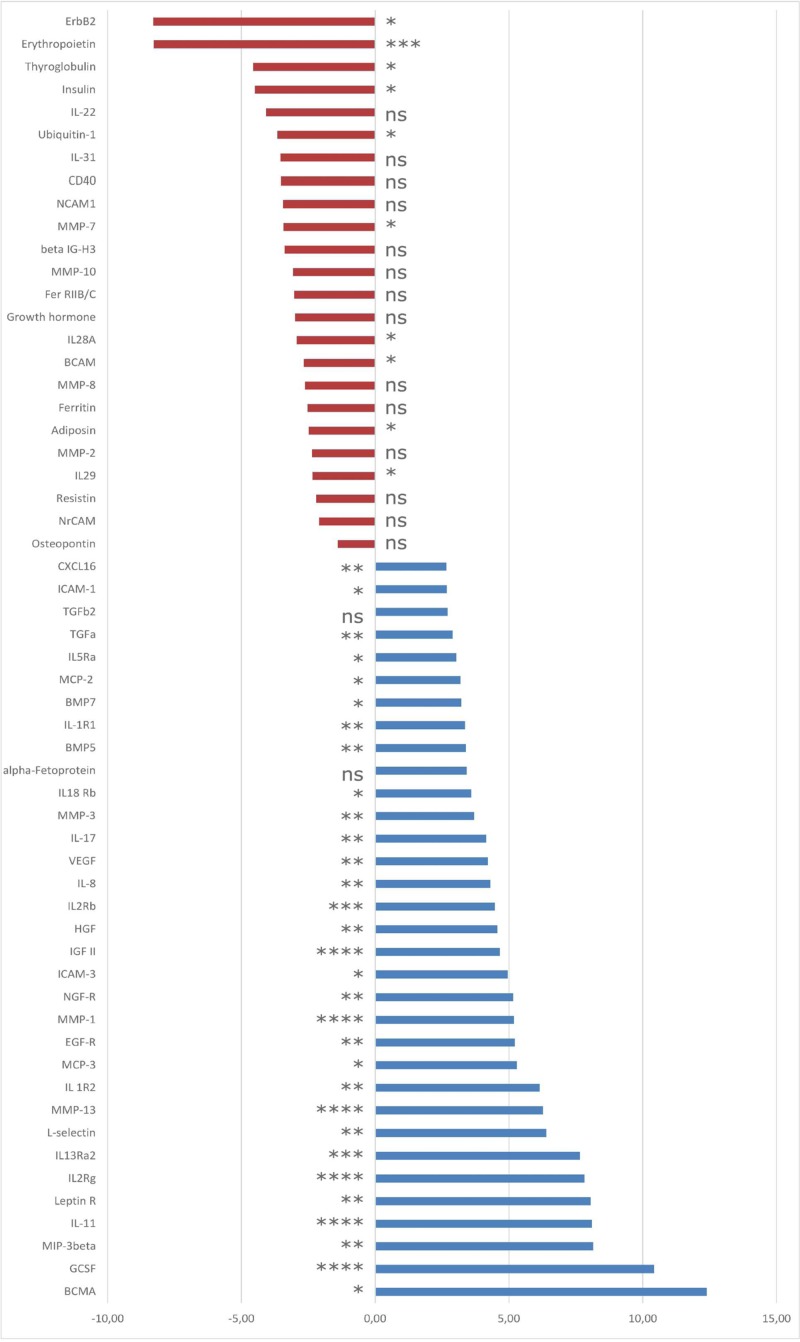
Fibrotic mechanisms following trabeculectomy, differentially expressed genes/proteins Selection of gene names of the proteins with significantly diminished (red) or increased (blue) expression in the AH of POAG patients after failed trabeculectomy compared with patients with successful surgery outcome. Data are presented as mean ± S.D. Levels of significance: *P≤0.05; **P≤0.01; ***P≤0.001; ****P≤0.0001; ns, not significant.

The cytokine antibody arrays identified 274 proteins in the AH samples. A comparison between patients with failed and successful trabeculectomy surgeries revealed different protein expression measurements in AH. One hundred and three of the identified proteins were significantly up-regulated in the failure group compared with the successful group, while 27 proteins were significantly down-regulated. Our study focused on the most important cytokines for fibrosis processes and the ones with the largest fold changes (Figure 2). In the following, the classification of proteins into functional groups was based on expert knowledge.

### Proteins with profibrotic properties

Failure of trabeculectomy is usually correlated with scar formation and fibrosis. We found many proteins up-regulated in AH from patients after failed trabeculectomy that are known to be involved in fibrosis. Among them, we found epidermal growth factor receptor (EGFR) with five times higher expression and VEGF (four times higher expression), compared with the group where surgical outcome was successful. TGF-β2 expression showed a 2.71 fold increase in comparison with the successful surgery outcome group, while bone morphogenic protein (BMP) 7, another member of the TGF family, showed 3.21 fold increase. Amid other up-regulated proteins we found nerve growth factor receptor (NGF-R) (5.16 fold increase) and hepatocyte growth factor (HGF) (4.56 fold increase). On the other hand, human EGFR 2 (HER2, also known as ErbB2) (8.3 fold decrease), erythropoietin (8.28 fold decrease), thyroglobulin (4.57 fold decrease), neural cell adhesion molecule 1 (NCAM1) (3.45 fold decrease), growth hormone (3.0 fold decrease) and basal cell adhesion molecule (BCAM) (2.68 fold decrease) were notably down-regulated compared with patients with successful surgery outcome.

### Proteins with inflammatory properties

The results indicate that almost 50% of up-regulated proteins from AH of the failed surgery group were linked to stimulation of inflammatory processes. Wound healing after glaucoma surgery is strongly supported by inflammation. We observed 12-times higher expression of B-cell maturation antigen (BCMA) and 10-times higher expression of granulocyte colony-stimulating factor (GCSF). IL-11, IL-13, IL-8, IL-1 and monocyte chemotactic protein (MCP) were also among the up-regulated cytokines, with expression up to eight-times higher compared with the successful surgery group. Overall, 49 proteins were up-regulated. In turn, six proteins (IL-22, IL-31, cluster of differentiation (CD) 40 (CD40), IL-28A, IL-29, osteopontin) which are involved in inflammatory processes were down-regulated in the failed surgery group, in comparison with the successful group.

### Proteins with adhesion properties

Our analysis showed up-regulation of certain adhesion molecules in AH from patients after failed trabeculectomy. Many of these are proteins that are responsible for the adhesion of inflammatory cells. L-selectin was six fold higher in the failed surgery group compared with the successful surgery group. Intercellular adhesion molecules 1, 2 and 3 (ICAM1, ICAM2, ICAM3), CD166 antigen, carcinoembryonic antigen-related cell adhesion molecule 1 (CEACAM1), stromal cell-derived factor 1 (SDF1), C–X–C motif chemokine 5 (CXCL5) and phosphatidylinositol-glycan biosynthesis class F protein were also markedly up-regulated with fold changes up to 4.95. Down-regulated adhesion molecules were β-IG-H3 (3.08 fold decrease) and neuronal cell adhesion molecule (NrCAM) (2.10 fold decrease).

### Proteins with apoptotic properties

Additionally, several proteins involved in apoptotic processes were significantly up-regulated in the AH of the failed surgery group compared with the successful group. The average fold change of these proapoptotic proteins was 2. Identified proteins were TRAIL receptor 4 (TRAILR4), FAS receptor (FasR), human soluble tumor necrosis factor receptor I (sTNF-RI), fas ligand (FasL), osteoprotegrin (OPG) and tumor necrosis factor receptor superfamily member 18 (TNFRSF18).

We also discovered that in AH of patients with failed trabeculectomy, MMP-1, MMP-3 and MMP-13 were significantly up-regulated, whereas MMP-2, MMP-7, MMP-8 and MMPs-10 were down-regulated.

Finally, all proteins detected in AH from patients were analyzed using *ExprEssence* based on a STRING network. The condensed network of Figure 1 reflects many previously known mechanisms of fibrosis, including interactions between cytokines. One of the most important mechanisms are the interactions of VEGF (which plays a key role in the condensed network) with many pro-fibrotic cytokines and growth factors, based on a consistent up-regulation together with TGF-β1, MMP-1, IL-8, EGFR, HGF, leptin and chemokine ligand 2. Another protein that was highlighted as being up-regulated in cases of failed trabeculectomy is IL-8, which interacts with VEGF and EGFR and is strongly up-regulated by CXCL1, HGF and IL-1a.

## Discussion

The present study aimed to identify cytokine/chemokine profiles of the AH of POAG patients, correlated with the early fibrosis processes after trabeculectomy. The early failure of trabeculectomy as defined by two needlings within the first 3 months and IOP > 21 mmHg with medications versus the early success as defined by an IOP within the range of the episcleral venous pressure (that is, IOP < 13 and > 8 mmHg) without medications is expected to give the most pronounced differences in the cytokine/chemokine profiles and may help to identify some of the key players of fibrosis. The present study was not designed to identify differences in the cytokine/chemokine profiles between glaucoma patients and healthy people.

Our results suggest that there are notable differences in the expression of many proteins in the AH of patients after early failed versus early successful trabeculectomy. In our findings we observed a large number of up-regulated and down-regulated proteins correlated with fibrotic processes, which were already suggested in the literature to play a key role in trabeculectomy outcome.

One of the proteins that was strongly up-regulated in our study was VEGF (4.21 fold). This cytokine had many interactions with other proteins identified by *ExprEssence* analysis (Figure 1). Besides its known role in neovascularization, many publications demonstrated a significant involvement of VEGF in fibrotic processes in a variety of organs [21,22], stimulating the growth of vascular endothelial cells and possibly taking part in wound healing after trabeculectomy [23,24]. VEGF was also reported to be elevated in AH of POAG and neovascular glaucoma patients [7]. Another report showed that VEGF plays a key role in activating TGF-β1 expression, especially the Smad/Snail pathway after glaucoma surgery, noting that it was significantly elevated after trabeculectomy [25].

We also found a strong up-regulation of VEGF and TGF-β in the failed surgery group in comparison with the successful surgery group. *ExprEssence* identified their strong interaction, confirming the findings of Park et al. [25], where TGF-β was already described as a fibrosis-stimulation cytokine. TGF-β promotes fibroblasts contraction, proliferation and migration [26]. It is a mediator of matrix proteins [27,28], fibronectin [29], collagens [30] and proteoglycan secretion processes [31]. It down-regulates proteolytic enzymes that are responsible for the degradation of matrix proteins and the turnover of the ECM [30,32]. Isoforms of TGF-β activate cell surface TGF-β serine/threonine kinase receptors which are connected to signal transduction networks, such as mitogen-activated protein kinase (MAPK)/extracellular signal-regulated kinase (ERK), p38Mapk, C-Jun-N-terminal kinase (JNK) and Smads [7]. As mentioned above, the Smad pathway was already described to be deeply involved in stimulating fibrosis induced by TGF-β. Cordeiro et al. [33] demonstrated that anti-TGF-β2 antibodies suppressed scar formation after filtering surgery in rabbits and glaucoma patients, resulting in longer bleb survival compared with untreated controls. Another study reported that a more desirable bleb development in POAG patients was among those with normal TGF-β2 levels in AH compared with those with higher ones [34].

As mentioned before, almost half of the proteins that we found to be up-regulated after failed trabeculectomy were connected to immune/inflammation processes. Both increased cytokine expression and tissue injury after surgical management lead to activation of fibroblasts, neutrophils and macrophages. Inflammatory cells infiltrate the wound directly after surgery and start to secrete pro-inflammatory cytokines, leading to inflammation and fibrosis [7]. One of the latest publications compared AH from glaucoma and cataract patients, demonstrating an up-regulation of cytokines correlated to innate immune processes among glaucoma patients compared with control groups. The authors observed an up-regulation of many proteins in AH from glaucoma patients, which were mostly related to innate immunity, for example higher values of CD14 and CD163, which are monocyte/macrophage markers [35]. In accordance, we also observed changes in values of CD14 (2.01 higher expression) in the failed surgery group compared with the successful surgery group.

Many of the cytokines with increased expression measurements in our study (IL-1α, IL-1β, IL-6, TNF-α, FasL) were already described as being elevated in glaucoma patients [36]. Additionally, IL-1 and IL-6 were also described to be up-regulated in fibrotic processes in other organs [37,38]. In accordance with already published data, Il-22 (which was described to have antifibrotic properties [7]) was strongly down-regulated in our group of patients with failed surgery outcome. This cytokine is an IL-10 family member, which was also shown to have an anti-inflammatory effect [39,40].

MCP-1 levels in AH of glaucoma patients were already described as a very relevant prognostic factor for trabeculectomy outcome [41]. In our protein examination we found that MCP family cytokines (MCP-2 and 3) were highly up-regulated in patients with failed trabeculectomy outcome, underlining this result. These cytokines can activate macrophage infiltration into local tissue [42] as well as leukocytes, which leads to excessive inflammatory and fibrotic responses [41]. It was already found that elevated MCP-1 levels in tears [43] and in AH [41] among patients with glaucoma are correlated with a higher tendency to postoperative scarring in an early phase after surgery, including trabeculectomy. MCP-1 was also shown to be a pro-fibrotic agent in other organs [44–46]. Additionally, an involvement of MCP-2 [47] and MCP-3 [48] in glaucoma and its treatment were described.

MMPs are one of the most characteristic proteases that are responsible for ECM degradation. Apart from wound healing processes they are also involved in embryogenesis, bone growth and angiogenesis [49]. It was already suggested they can play a role in excessive scarring processes. Observations using a rabbit model and the MMP inhibitor, GM6001, showed a reduction in scarring after filtering glaucoma surgery [50]. We observed a significant down-regulation of some MMPs. One of the most important is MMP-2, which is responsible for degradation of collagen (IV–VI, X) elastin and fibronectin [49]. This protease was 2.37-times down-regulated in patients after failed trabeculectomy, suggesting it plays a key role in ECM levels during excessive wound healing processes.

Based on the literature, the groups of proteins discussed above play a significant role in the fibrotic processes after trabeculectomy surgery. In our study we found a number of strongly up-regulated cytokines, already described in the literature as pro-fibrotic. Moreover, activated inflammatory cells infiltrate the post-surgery wound and secrete pro-inflammatory cytokines which also lead to fibrosis. We observed up-regulation of certain apoptotic markers in the group of patients after failed trabeculectomy, indicating that programmed cell death occurs in this case. It is known that myofibroblasts undergo apoptosis at the end of the healing process [51]. This process is strongly influenced by the cytokine TGF-β which inhibits programmed cell death in myofibroblasts [52] and on the other hand induces the transformation of fibroblasts into myofibroblasts. Our observations indicate a misbalance between these processes which leads to a continuous activation of fibroblasts by increased levels of TGF-β and therefore an uninterrupted but extenuated process of apoptosis in myofibroblasts, which finally results in fibrosis. Additionally, glaucoma and increased IOP can lead to neuronal cell death due to oxidative stress [53,54]. Peroxireduxin 2 and 6, and superoxide dismutase, are proteins responsible for antioxidant protection, which were already described as down-regulated among glaucoma patients. The same authors also observed down-regulation of proteins connected to neuronal physiology [35]. Apoptotic cell death in RGCs was already described in an experimental glaucoma model in monkeys [55] and in humans suffering from glaucoma [56]. Increasing IOP levels after failed trabeculectomy can lead to the progression of apoptosis in RGCs and to an accumulation of apoptotic markers. Taking into consideration that the vitreous is more fluidic with age due to a decrease in the elasticity of the hyaluronic acid and that a posterior vitreous detachment (PVD) is more prominent in elderly people (who form the majority of glaucoma patients), it is conceivable that the apoptotic factors found in our study could have their origin also in RGCs. Furthermore, an increase in the prevalence of PVD was demonstrated in glaucoma patients in comparison with healthy subjects [57] which facilitates diffusion of apoptotic factors to the AH. On the other hand, most of the cytokines and factors identified in our study are correlated to fibrosis, inflammation, wound healing and ECM modulation. Therefore, we assume that the apoptotic factors also have their origin in the apoptosis of myofibroblasts. To finally clear this phenomenon further investigations are necessary.

There are some limitations of the study: first, results were based on one experimental approach (antibody arrays); second, the sample size of eight patients is quite small; and third, the study had a retrospective design. This might limit the conclusions of the study.

## Conclusion

This pilot study indicated alterations in the expression of various proteins in AH of early successful and failed trabeculectomy surgeries. We demonstrated that many of the differences in expression of affected proteins are linked to fibrotic processes. These findings suggest that a negative outcome of trabeculectomy is strongly related to the stimulation of fibrotic processes. These results could be used to identify substances such as inhibitors which may be able to limit early fibrotic mechanisms in specific ways and thus to avoid surgical interventions like bleb needling in the early postoperative period. Further studies on cytokine/chemokine profiles of AH after trabeculectomy are needed to identify further fibrotic mechanisms, to validate the findings, and to subsequently improve early as well as late trabeculectomy outcomes.

## Supporting information

**Supplementary Data S1:** 

**Supplementary Data S2:** 

## Abbreviations

AH: aqueous humor
ANOVA: Analysis of Variance
CD: cluster of differentiation
ECM: extracellular matrix
EGFR: epidermal growth factor receptor
FAS: Tumor Necrosis Factor Receptor Superfamily Member 6
FasL: fas ligand
HGF: hepatocyte growth factor
IL: Interleukin
IOP: intraocular pressure
MCP: monocyte chemotactic protein
POAG: primary open-angle glaucoma
PVD: posterior vitreous detachment
RGC: retinal ganglion cell
STRING: search tool for recurring instances of neighboring genes
TGF-β: transforming growth factor β
TNF-α: tumor necrosis factor-α
TRAIL: Tumor Necrosis Factor Related Apoptosis Inducing Ligand
VEGF: vascular endothelial growth factor
